# European citizens' use of E-health services: A study of seven countries

**DOI:** 10.1186/1471-2458-7-53

**Published:** 2007-04-10

**Authors:** Hege K Andreassen, Maria M Bujnowska-Fedak, Catherine E Chronaki, Roxana C Dumitru, Iveta Pudule, Silvina Santana, Henning Voss, Rolf Wynn

**Affiliations:** 1 Norwegian Centre for Telemedicine, University Hospital of Northern Norway, Tromsø, Norway; 2 Department of Family Medicine, Wroclaw Medical University, Wroclaw, Poland; 3 Foundation for Research and Technology, Heraklion, Greece; 4 Chair of Medical Informatics, Friedrich-Alexander-University Erlangen-Nürnberg, Erlangen, Germany; 5 Health Promotion State Agency, Riga, Latvia; 6 Universidade de Aveiro, Aveiro, Portugal; 7 Danish Centre for Health Telematics, Odense, Denmark; 8 Department of Clinical Psychiatry, University of Tromsø, Tromsø, Norway

## Abstract

**Background:**

European citizens are increasingly being offered Internet health services. This study investigated patterns of health-related Internet use, its consequences, and citizens' expectations about their doctors' provision of e-health services.

**Methods:**

Representative samples were obtained from the general populations in Norway, Denmark, Germany, Greece, Poland, Portugal and Latvia. The total sample consisted of 7934 respondents. Interviews were conducted by telephone.

**Results:**

44 % of the total sample, 71 % of the Internet users, had used the Internet for health purposes. Factors that positively affected the use of Internet for health purposes were youth, higher education, white-collar or no paid job, visits to the GP during the past year, long-term illness or disabilities, and a subjective assessment of one's own health as good. Women were the most active health users among those who were online. One in four of the respondents used the Internet to prepare for or follow up doctors' appointments. Feeling reassured after using the Internet for health purposes was twice as common as experiencing anxieties. When choosing a new doctor, more than a third of the sample rated the provision of e-health services as important.

**Conclusion:**

The users of Internet health services differ from the general population when it comes to health and demographic variables. The most common way to use the Internet in health matters is to read information, second comes using the net to decide whether to see a doctor and to prepare for and follow up on doctors' appointments. Hence, health-related use of the Internet does affect patients' use of other health services, but it would appear to supplement rather than to replace other health services.

## Background

There is a considerable demand for health-related information in the population, and the Internet is gaining ground as a central source of such information [1,2]. In the US, studies have found that between 56 % and 79 % of Internet users seek health information [3-6]. Starting out as a grassroots phenomenon much used by individual patients operating on their own and often offered by idealists as well as by commercial interests, Internet health sites and other electronic communication tools targeting patients are now important policy instruments for both public and private health providers. In recent years, we have seen national health authorities beginning to focus on e-health services such as electronic health cards, electronic patient records and health portals, including the English NHS Direct Online, the German Telematic Platform, and the Danish Sundhed.dk. In the medical community, expectations about the Internet are mixed. On one hand, the Internet has been described as having the potential to empower patients and stimulate patient participation [7-10]. On the other hand, potential dangers such as the dissemination of inaccurate information and inappropriate use have been stressed [11-13]. Earlier European studies have shown that the use of the Internet for health purposes varied in different parts of Europe [14,15]. As the dissemination of e-health services is growing along with general Internet use, there is a need to improve our knowledge on how these services are used, by whom and with what consequences. Two research questions were pursued in the present study; Do the users of Internet health services differ from the general population with respect to health and demographic variables? And, which health related Internet activities are most common? Further, we investigated citizens' expectations concerning the provision of e-health services by doctors.

## Methods

A study group of 20 researchers designed a questionnaire for computer-assisted telephone interviews (CATI). The questionnaire was piloted with 100 individuals to ensure the comprehensibility of the wording and internal validity. It was designed in English and translated into the other languages by means of the dual focus approach [16]. This approach differs from the translation-back translation method in that it focuses on conceptual equivalence as well as on equivalence in wording and grammar. The aim is to reduce potential cultural bias in the questionnaire. The survey was conducted during the period October to November 2005. Random digit dialling in stratas ensured a randomised representative sample of the populations (age group 15 – 80 years) of seven European countries. The telephone penetration was estimated to be close to 100 % in Norway, Denmark, and Germany. In Poland it was estimated to be 63 %, in Latvia 93 %, in Greece 87 %, and in Portugal 65 %. Mobile phone numbers were included in Norway, Denmark, Germany, and Latvia. Sampling continued until we had approximately 1000 completed interviews from all countries, except Portugal where 2000 interviews were conducted as health- related Internet use was expected to be low. Calculating a response rate is difficult when this sampling procedure is used, as a required number of responses is set before sampling starts, and sampling actually continues until the required number is obtained. The polling agencies conducting the interviews were instructed to follow standard procedures relating to contacting a replacement if a person originally selected for interview was unavailable (i.e. because of incorrect phone number, not answering the phone, not at home, or unwilling to participate). Nevertheless, we lack accurate data from all agencies relating to the number of people who were contacted in order to achieve the final number of completed interviews. A population weight was used to correct for differences in the sizes of the countries' populations for total estimates and logistic regression. No variables had more than 5% missing data. National ethics committees from all countries were informed and had no objections to the survey. We analysed the data by performing descriptive statistics and logistic regression analysis. SPSS version 12.0 was used for all analyses.

## Results

The total sample consisted of 7934 respondents; out of these 4714 reported that they were Internet users. After weighting for population size, we had a total sample of 7903, of which 4906 were Internet users.

Before weighting, we calculated the proportion of Internet health users in each country (Table 1). Health-related use of the Internet was most frequent in the Northern countries, with Denmark (62 %), and Norway (59 %) topping the list, followed by Germany (49 %). The Eastern countries, Poland and Latvia, reported 42 % and 35 % health-related use of the Internet respectively, while the Southern countries had the lowest proportion of Internet health users with 30 % in Portugal and 23 % in Greece. In the sub-sample of Internet users, the differences between the countries were smaller, but a chi-square test showed that the differences between the Northern (74 % Internet health users), East-European (72 %) and Southern countries (60 %) were significant (χ^2^_(2,4714) _= 88, 5, p < 0.001), despite the high score in Poland (79 %).

**Table 1:** Internet health users in 7 European countries.

**Country**	**Total sample**	**Internet Users**		**Internet Health Users**		
			
		**Count**	**% of Total sample**	**Count**	**% of Total sample**	**% of Internet Users**
Denmark	960	777	81 (78–83)	595	62 (59–65)	77 (74–80)
Germany	974	670	69 (66–72)	473	49 (45–52)	71 (67–74)
Greece	1000	422	42 (39–45)	229	23 (20–26)	54 (49–59)
Latvia	1000	534	53 (50–57)	346	35 (32–38)	65 (61–69)
Norway	972	778	80 (78–83)	577	59 (56–62)	74 (71–77)
Poland	1027	545	53 (50–56)	428	42 (39–45)	79 (75–82)
Portugal	2001	988	49 (47–52)	598	30 (28–32)	62 (59–65)

Total count and % in the populations and among Internet-users with (95% Confidence Intervals)

In the joint population of the seven countries, a total of 44 % (71 % Internet users) reported having used the Internet for health purposes (Table 2). In the general population, men were the most active health users on the Internet (47 % men, 42 % women). However, when Internet access was controlled for and we concentrated on those who were online, women tended to use the Internet more for health purposes than men (75 % women, 68% men). In the total sample, the youngest age group (15–29 years) was more concerned with looking for health information (63 %). Among the Internet users, the 30–44 age group included the most active health users (74 %). Regression analysis revealed that people with higher education and those working in a white-collar profession or not working at all tended to use the Internet more for health purposes. The same applied to those who had visited a general practitioner during the past year and to those who suffered from long-term illness or disability. Subjective assessment of health status had an opposite impact on health-related Internet use in the total sample; those who reported their health to be poor used the Internet less for health purposes than did other respondents. In the total sample, being next of kin to an ill person also increased the likelihood of using the Internet for health purposes, while this correlation did not prove to be significant in the sub-sample of Internet users.

**Table 2:** Factors that affect health-related use of the Internet^1^

	**Total sample**					**Internet users**				
	**Total**	**count**	**%**	**Odds ratio (95% CI)**		**Total**	**Count**	**%**	**Odds ratio (95% CI)**	
		
**Gender**										
M	3457	1630	47		1	2401	1630	68		1
F	4441	1866	42	***	0,80 (0,72–0,89)	2500	1866	75	*	1,17 (1,03–1,34)
**Age group**										
15–29	2045	1284	63		1	1819	1284	71		1
30–44	2335	1284	55	***	0,59 (0,52–0,68)	1727	1284	74	*	1,25 (1,06–1,48)
45–59	1875	737	39	***	0,34 (0,29–0,39)	1055	737	70		0,99 (0,82–1,20)
60 +	1644	191	12	***	0,08 (0,07–0,10)	299	191	64	***	0,61 (0,47–0,80)
**Completed education^2^**										
Below A-Level	2149	520	24		1	820	520	63		1
A-Level	4276	2076	49	***	2,18 (1,92–2,48)	2885	2076	72	***	1,42 (1,20–1,69)
Above A-Level	1473	900	61	***	3,98 (3,36–4,70)	1195	900	75	***	1,88 (1,52–2,32)
**Work status**										
No paid work	4142	1495	36		1	2030	1495	73		1
Blue-collar position	1443	574	40	*	0,83 (0,72–0,96)	904	574	64	***	0,61 (0,51–0,74)
White-collar position	2311	1426	62	***	1,60 (1,40–1,83)	1966	1426	74	*	0,81 (0,68–0,95)
**Visits to the GP last year**										
0	1188	498	42		1	760	498	66		1
1–5	4502	2110	47	***	1,33 (1,16–1,54)	3015	2110	70	*	1,24 (1,04–1,48)
More than 5	2041	823	40	***	1,58 (1,33–1,87)	1037	823	79	***	1,94 (1,55–2,41)
**Assessment of own health status**										
Good	5263	2686	51		1	3770	2686	71		1
Fair	2173	705	32	***	0,70 (0,61–0,79)	988	705	71		0,94 (0,80–1,11)
Poor	448	102	23	***	0,53 (0,40–0,69)	139	102	71		0,83 (0,55–1,25)
**Current long-term illness or disability**										
No	6477	2872	44		1	4134	2872	82		1
Yes	1421	624	44	***	1,60 (1,38–1,85)	766	624		***	1,73 (1,40–2,15)
**Long-term illness or disability in the family**										
No	4160	1773	43		1	2413	1773	74		1
Yes	3738	1723	46	*	1,14 (1,02–1,27)	2487	1723	69		0,92 (0,80–1,06)
**Total sample**	7903	3496	44			4901	3496	71		

^† ^A-level education is equivalent to completed secondary school

‡ Variables included in logistic regression: Gender, age, education, employment status, number of visits to GP, subjective assessment of health status, personal long-term illness or disability diagnosis, long-term illness or disability diagnosis in the family

*Significant at p < 0,05 **Significant at p < 0,005 ***Significant at p < 0,001

Table 3 shows that one of the most frequent consequences of use was a feeling of reassurance or relief (19 % of the sample). Feelings of anxiety were reported by 10 %. When asked how important they considered the Internet to be as a source of health information, 3141 of the respondents, 40 % of the total sample (53 % of the Internet users), reported it to be important or very important (Table 4). The corresponding figure for face-to-face interaction with health professionals was 6469 respondents, that is, 82 % of the total sample (81 % of the Internet users). Table 5 presents the importance of different e-health services in the choice of a doctor in the total population and among Internet users.

**Table 3:** E-health activities and consequences in the total sample and among Internet users^3^.

*Activities (Have you used the internet to...)*	**Count**	**% in total sample (N = 7903)**	**% among Internet users (N = 4906)**
Interact with web doctor/health professional you have not met	1485	19	30
Approach family doctor or other known health professionals	325	4	7
Self-help activities	1325	17	27
Order medicines/health products	1016	13	21
Read about health or illness	2567	33	52
Decide whether to see a doctor	2254	29	46
Prepare for an appointment	1830	23	37
Look up information after an appointment	2139	27	44
*Consequences (Has information you obtained from the Internet led to any of the following)*			
Feelings of anxiety	754	10	15
Feelings of reassurance or relief	1464	19	30
Willingness to change diet/lifestyle habits	1611	20	33
Suggestions/queries about diagnoses	1612	20	33
Change of medicine without consulting a health professional	192	2	4
Making, cancelling or changing a doctor's appointment	445	6	9

^1^Sample weighted for population size.

**Table 4:** How people value the importance of different health information channels.

**Health information channel**	**Total sample (N = 7903)**		**Internet users (N = 4906)**	
		
	**count**	**%**	**count**	**%**
Face to face contact with a health professional	6469	82	3993	81
Family and friends	5032	64	2985	61
Books/encyclopedias	4821	61	3098	63
TV/Radio	4770	40	2734	56
Pharmacies	4735	60	2755	56
Newspapers/magazines	4497	57	2667	54
Courses and lectures	2735	56	1774	36
The Internet	3141	40	2607	53

^1 ^Sample weighted for population size. Included in the table are those who answered 4 or 5 on a 5-point scale where 5 was very important

**Table 5:** Importance of different e-health services in the choice of a doctor in the total population and among Internet users.

**Doctors' facilities**	**Total sample (N = 7903)**		**Internet users (N = 4906)**	
		
	**count**	**%**	**count**	**%**
E-mail communication	2738	35	2228	45
E-mail prescriptions	1774	22	1380	28
Order/change appointments online	2658	34	2099	43
Doctor's office has website	3107	39	2343	48
Reminders via SMS	2744	35	1914	39
Access to own electronic patient record	2873	36	2175	44
Cost of services	4305	55	2654	54
Information on the doctors' practice	4424	56	2902	59
Recommendation by others	4852	61	3232	66
General accessibility	5867	74	3826	78

^1 ^Sample weighted for population size. Included in the table are those who answered 4 or 5 on a 5-point scale where 5 was very important

## Discussion

Some aspects of the present study should be improved in a follow up study. As mentioned in the methods section, we were unable to calculate an exact response rate due to lacking data from the polling agencies. Even though the number of respondents was high and even though lacking responses to phone calls may be due to many factors, the response rate is of importance to the assessment of the validity of studies such as the present. The telephone penetration in Poland is quite low, which may be of importance to the calculation of the use of e-health services. A future study should therefore include a proportion of mobile phone users in the Polish sample. Income was not included as a variable in the present study. Although education and profession are variables of importance to socio-economic status, adding an income variable could give an even better understanding of the respondents' socio-economic background.

Use of Internet health services varies with country of residence. The North European countries and Poland topped the list, while we found the South European countries at the bottom. As the differences are significant within the sub-sample of Internet users as well, they may not be associated solely with the degree of general Internet access. Two explanations are possible: first, cultural differences, such as preoccupation with health and illness together with other factors, such as the number of accessible web-sites in local languages and the quality and accessibility of general health services, may be of importance [12]. Second, it may be that the Internet user group in the Southern countries is dominated by early adopters, and that the interest in health issues is lower in this group than it is in the general population. If so, we might assume that geographical differences will even out as access becomes more evenly distributed in the national populations.

In the sub-sample of Internet users, women reported more health-related use. This finding is in line with that reported by some studies from the US [1,3,17], that female Internet users are more interested in health-related issues. The youngest age group comprises the most ardent Internet users, but it is the young adults and the middle aged who take most interest in health information once they are online. A plausible explanation is that we find a large proportion of family caregivers in this group. Having completed higher education has previously been found to be associated with higher use of the Internet for health purposes [1,3], a finding which this study confirms. Having a white-collar position usually means longer education; thus it is not surprising that this group are more active Internet health users. We also found a high level of health-related use of the Internet among people who did not have paid work, a possible explanation for this being that students form an important part of this group.

Those who assessed their own health status as poor tended to use the Internet less than others to get health information. However, medical indicators of health, such as a current diagnosis of long-term illness or disability, and a high number of visits to the GP, indicate a higher level of health-related use of the Internet. Hence, we find that those who suffer from illness but who nevertheless feel that they are in good health use the Internet most for health purposes. Concern has been expressed that there might be some patients who feel they are too ill or who do not have the resources to use the Internet [18]. Our study indicates this might be the case. It is important to keep such differences between patient groups in mind when future e-health services and strategies are developed, in order not to widen the gap between the well off and the less well off in society [19].

Our study confirms that the main health-related activity on the Internet is information seeking [1,2]. However, a considerably higher number than previously reported [3] used the Internet as a communication channel. Among Internet users, 27 % had participated in forums or self-help groups and 30 % had interacted with health professionals. This indicates that other health-related activities on the Internet are becoming increasingly important, and that e-health services have already become an important part of health care for many people, as has also been suggested by other studies [20].

The possible relation between health related Internet usage and peoples' use of other health services has been given attention in later years [9,21,22]. In our study, three findings are of particular interest with regard to this topic: Only 6 % claim they have made, cancelled or changed a doctor's appointment based on health related Internet activity. Second, we found that people primarily use the Internet for general reading. And third, that approximately a quarter of the respondents actually use the Internet to prepare for or follow up a doctor's appointment. Hence we conclude that the Internet is used as a supplement to the ordinary health services rather than as a replacement. Another finding that supports our conclusion is the relatively low number of respondents (40 %) who claimed that the Internet was an important channel for health information (Table 4). Face-to-face contact with a health professional was considered important by almost twice as many, 79 %. However, even if our study shows the Internet is used as a supplement, we also see indications that health related Internet activity affect the populations' use of traditional medical services. One third of the Internet users have brought with them to their doctor suggestions or queries on diagnosis after surfing the net for health information. And almost half of the Internet users claim they have used the Internet to decide whether they need to see a doctor. As the number of European general practitioners offering e-health services is still low, we are not surprised that only 4 % of respondents reported that they had approached their family doctor via the Internet.

It was twice as common to feel reassured as it was as to feel anxious after using the Internet for health purposes. Hence, our study supports the idea that the populations' use of Internet health information is more likely to have a beneficial than a negative influence on individual health experiences [21].

A sign of the increasing importance of the Internet in citizens' health management is that about a third of the respondents stated that the doctor's provision of e-health services was of importance when choosing a new doctor. The differences between the expectations of Internet users and the general population, as presented in Table 5, support the idea that it is likely there will be an increasing demand for provision of e-health services by primary care and hospital services as more and more Europeans obtain Internet access [23].

## Conclusion

The Internet is becoming an important source of health information and a potential e-health channel for European citizens. The users of Internet health services differ from the general population when it comes to health and demographic variables. The most common way to use the Internet in health matters is to read information, second comes using the Internet to decide whether to see a doctor and to prepare for and follow up on doctor's appointments. Hence, health-related use of the Internet does affect patients' use of other health services, but it would appear to supplement rather than to replace ordinary health services. It is twice as common for users to feel reassured after accessing the Internet for health purposes as it is to experience anxiety. Doctors are likely to find that patients expect them to offer e-health services. Future strategies should ensure that e-health services are implemented with care, in order not to consolidate or create new inequalities in health care. It will be of great importance to follow up on studies of European citizens' use of e-health.

## Competing interests

The author(s) declare that they have no competing interests.

## Authors' contributions

HKA contributed to conception and design, acquisition of data, analysis and interpretation of data, and drafting and revising the manuscript. CEC contributed to conception and design, acquisition of data, analysis and interpretation of data, and drafting and revising the manuscript. SS contributed to conception and design, acquisition of data, analysis and interpretation of data, and drafting and revising the manuscript. HV contributed to conception and design, acquisition of data, analysis and interpretation of data, and drafting and revising the manuscript. RW contributed to conception and design, acquisition of data, analysis and interpretation of data, and drafting and revising the manuscript. MMBF contributed to conception and design, acquisition of data, and drafting and revising the manuscript. RCD contributed to conception and design, acquisition of data, and drafting and revising the manuscript. IP contributed to conception and design, acquisition of data, and drafting and revising the manuscript. All authors read and approved the final manuscript.

## Pre-publication history

The pre-publication history for this paper can be accessed here:


